# Completing Death Certificates in Hospital Setting: What Can Go Wrong, Will Go Wrong

**DOI:** 10.1177/19253621251343662

**Published:** 2025-05-27

**Authors:** Cécile M. Woudenberg-van den Broek, Annemiek M. Vuurens, Paul L.P. Brand, Walther N.K.A. van Mook, Ralph K.L. So, Wilma L.J.M. Duijst-Heesters

**Affiliations:** Department of Criminal Law and Criminology, Faculty of Law, 5211 Maastricht University Role: A, B, C, D, E, 1; 8122 Netherlands School of Public and Occupational Health Role: B, C, D, E; Isala Academy, Department of Medical Education and Faculty Development, 8772 Isala Hospital; Department of Intensive Care Medicine, Maastricht University Medical Center+; Department of Intensive Care, Albert Schweitzer hospita Role: B, C, D, E, 5, 6; Department of Criminal Law and Criminology, Faculty of Law, 5211 Maastricht University Role: A, B, C, D, E, 4, 6; Wenckebach Institute for Medical Education, University of Groningen and University Medical Centre Groningen Role: B, C, D, E, 5, 6; School of Health Professions Education (SHE), 5211 Maastricht University; Academy for Postgraduate Training, Maastricht University Medical Center+ Role: B, C, D, E, 5, 6

**Keywords:** Forensic pathology, Forensic medicine, Cause of death, Death certification, Medical certificate of cause of death (MCCD), Hospital physicians

## Abstract

**Introduction:**

Of the 170 000 death certificates filled out annually in the Netherlands, approximately a quarter are completed by a hospital physician. From a legal and statistical point of view, it is important that the legally required forms, that is the certificate of death (CD), the medical certificate of cause of death (MCCD), and the envelope of the MCCD used for medical secrecy reasons are filled out correctly (ie, according to law) and accurately (ie, cause of death in line with medical files).

**Materials and Methods:**

For this study, 517 CDs and MCCDs were collected from three hospitals over a 5-month period. The CDs and MCCDs were analyzed to determine to what extent they met the formal (correctly, according to law) and material (accuracy of the cause of death as compared to medical file information) requirements.

**Results:**

In only 34 cases (6.7%) the hospital physicians fulfilled the formal requirements for completing both forms and the envelope of the MCCD. Sixteen cases were issued as natural deaths, although they were unnatural deaths. In 55 cases (11%) a phenomenon associated with death such as “no circulation” or “no breathing” was recorded as cause of death. Only 23 cases (4.5%) met the requirements of an accurate cause of death and correct completion of forms and envelope.

**Discussion:**

Hospital physicians rarely met the formal requirements when filling out the forms and corresponding envelope. Furthermore, the accuracy of the reported cause of death reveals potential for improvement.

**Conclusion:**

Correctness and accuracy in these forms are important not only from a legally administrative and criminal law point of view, but also for the robustness of public health epidemiology and the related funding of prevention of the established causes of death.

## BACKGROUND

In most countries, a physician fills out the appropriate forms when a person dies of an apparent natural death. In the Netherlands, these forms include the certificate of death (CD, called the A-form in the Netherlands), in which the attending physician declares to be convinced of a natural death, and the medical certificate of cause of death (MCCD, or B-form in the Netherlands) in which the attending physician reports the cause of death to Statistics Netherlands, the national agency with the statutory task of compiling statistics on a wide range of topics that are important to society and making the outcomes publicly available. As approximately a quarter of the 170 000 annually deceased die in a hospital (1), hospital physicians perform external postmortem examinations regularly. Previous studies have shown that physicians in general receive very little if any education on and training in performing external postmortem examinations (2–5). In two studies in which fictitious cases were presented to hospital physicians and general practitioners to assess how they acted, once their decision on the manner of death (ie, natural or unnatural death) was made, participants were inconsistent in their acts and thoughts regarding external postmortem investigations and lacked legal knowledge about these investigations (6,7). An earlier study of MCCDs in the Netherlands focused on the unnatural death as categorized by Statistics Netherlands (excluding traffic accidents) and showed that in approximately 28% of the unnatural deaths were reported as natural death by the attending physician (8). Formal requirements are important from a legal and administrative point of view. The importance of an accurate MCCD goes beyond statistics; the results of these forms contribute to a better understanding of public health issues and may affect the choice of publicly funded preventive measures.

Consequently, the aim of the present study was to analyse the correctness (ie, according to the formal requirements) and accuracy (ie, the cause of death was in line with the medical file) of the forms completed by hospital physicians in cases of assumed natural death. Furthermore, because medical specialists train residents in external postmortem examinations, we also analysed the difference in performance between medical specialists and residents in this matter.

### Setting: Dutch Legal System of Postmortem Examination

When a person dies in the Netherlands, the attending physician must perform an external postmortem examination (article 3 of the Burial Act, Wet op de Lijkbezorging). If an attending physician is convinced of a natural cause of death, that physician must fill out all the appropriate forms (article 7 p.1 and article 12a p.1 of the Burial Act). The physician declares to be convinced of a natural cause of death in the CD. In the MCCD, the physician states the actual cause of death according to the World Health Organization (WHO) system (9). The MCCD must be placed in a closed and sealed envelope for medical privacy.

If the attending is not convinced of a natural cause of death, he has to inform a forensic physician immediately (article 7 p.3 of the Burial Act), who will then take over the external postmortem investigation. If the forensic physician is convinced of a natural cause of death, he will fill out the same forms as the attending physician would have done. If he is not convinced of a natural cause of death, he will inform the prosecutor forthwith (article 10 of the Burial Act). Further investigation will then be considered in the form of forensic radiology, toxicology, or a forensic autopsy.

Furthermore, when the deceased is a minor (ie, under the age of 18), the attending physician must always inform the forensic physician of the death (article 10a of the Burial Act). The forensic physician will advise, based on the information given by the attending physician, whether a natural or unnatural death occurred or whether further investigation is required.

**Table 1** shows a description of the formal requirements for filling out the CD, the MCCD and the envelope.

**Table 1.** Formal Criteria According to Dutch Law for the CD, MCCD and Envelope for the MCCD.

Correctly filled out on CD	Correctly filled out on MCCD	Envelope of MCCD
Name of attending physician	Municipality of death	Last name of deceased (in case of a married person their own last name should be filled out and not their married or assumed name)
Place of work of attending physician	Location where the person died (ie, hospital, psychiatric hospital, nursing home, care home, other institution, home, other, and unknown)	Municipality of death
All first and middle names of deceased	Whether an autopsy has taken place, will take place, will not take place, unknown	Date of death
Last name of deceased (in case of a married person their own last name should be filled out and not their married or assumed name)	What further examinations have been or will be performed, such as histology, radiology, toxicology, etc	Should enclose MCCD and not the CD
Date of birth	Gender of deceased	Closed
Place of birth	Date birth	Signed on back to seal envelope
Place where the deceased was last registered	Date of death	
Date of death	Natural death-section with the cause of death through the 1a/b/c/2-system (the unnatural death-section of the form should never be filled out by an attending physician and is reserved for the forensic physician)	
Date of filling out the form	Length of time between onset of aforementioned disease and death	
Signature	Name of attending physician	
In case of a minor, the name of the forensic physician that the attending physician consulted and the date on which he did so	Corresponding address of attending physician	
	Date of filling out the form	
	Signature	

Abbreviations: CD, certificate of death; MCCD, medical certificate of cause of death.

The Netherlands have a system of postmortem examination where two medical doctors play a key role. These are the forensic physician and the forensic pathologist. The forensic physician is a specialized physician who performs external postmortem investigations in cases of (suspected) unnatural death and who writes reports on the assessment and interpretation of injury after forensic medical examination. The forensic pathologist is a physician specialized in pathology and subspecialised in forensic pathology and performs autopsies to provide cause of death at the request of the prosecution.

## METHODS

### General

This retrospective cohort study was carried out in three Dutch hospitals in various geographic regions: a university medical center (the Maastricht University Medical Centre+) and two general teaching hospitals (Isala Hospital in Zwolle and the Albert Schweitzer Hospital in Dordrecht). The death certificates, filled out by hospital physicians, of all the deceased in the hospitals during a period of five months between May 2021 and January 2024 were collected. The 5 months’ period was chosen in order to include at least 150 cases per hospital. Due to organizational issues, data gathering did not start simultaneously. All deceased adults and children were included. Deceased foetuses were excluded. Hospital morgue staff collected the forms and envelopes and scanned the CD, the back and front of the MCCD envelope and both sides of the MCCD, after opening the envelope. They also recorded whether the MCCD envelope was found closed or open.

The electronic medical records of the deceased were made available through the hospitals and were used to check the completed forms, particularly the MCCD. The cause of death, as recorded in the medical file of the deceased, was deduced from the last correspondence with the general practitioner, from the attending physician's case notes at the time of death, or from the medical file notes describing the hospital admission during which the patient died.

Incomplete cases were excluded, ie if one of the forms or the envelope was not (completely) scanned, if the scanned form was illegible or if the medical file could not be accessed for any reason.

A certified medical research database system, ResearchManager, was used to collect the information on an anonymous score form, one form per case. The information was gathered by two researchers (CW and AV). Most of the information gathered was factual. If uncertainties arose on the agreement between the medical records and the completed forms or on the interpretation of the cause of death as recorded on the form, they were resolved by discussing these cases with another forensic physician (WD) until consensus was achieved. To assess agreement between the researchers on their interpretation of the collected data, one in seven cases, randomly selected through an online random number generator, were scored by both researchers.

Data stored in the hospital's patient data management system were used to assess whether the forms were filled out by medical specialists or by residents.

The cases were checked to determine whether they met the formal and material requirements.

It was also noted whether the forms of the same case were completed by the same physician or by two different physicians. Since completion of a set of forms in a case by two different physicians is unlawful, these cases were excluded from analysis of the formal requirements and material requirements.

### Formal Requirements

Formal requirements as demanded by Dutch law are presented in **Table 1**. If one or more CD items were not filled out correctly, the CD was considered to not be filled out as required by law. The same was true for the MCCD and its envelope.

### Material Requirements

The MCCD was also checked for material criteria, that is, criteria on how a cause of death should be filled out according to the International Classification of Diseases, tenth edition (ICD-10) (9). When the noted cause of death was the actual cause of death and was in line with the medical file the MCCD was classified as having been filled out “accurately.”

The mistakes found when checking the completed forms against the material requirements were categorized as follows:

− Category A: unnatural cause of death wrongly declared as natural cause of death;
− Category B: a stated cause of death that is not a cause of death;
− Category C: a phenomenon of death as cause of death;
− Category D: a cause of death on the MCCD that cannot be found in the medical file;
− Category E: the cause of death found in the medical file is different from that on the MCCD, ie, such a discrepancy that it would change the overall ICD-10 category;
− Category F: no (major) discrepancies.

Examples per category can been seen in **Table 2**.

**Table 2.** Examples Per Category of Mistakes Found Regarding the Material Requirements.

Category	Description of category
Category A	Cause of death given as pneumonia after an acute subdural hematoma after a fall and trauma to the head. This should have been considered an unnatural cause of death where a forensic physician should have been consulted
Category B	Cause of death given as “resuscitation” or “progressive decline of age”
Category C	Cause of death given as asystole or respiratory arrest. These are phenomenon seen in death but not a cause as such
Category D	Cause of death stated on the MCCD as “myocardial infarction” or “pneumonia,” but no mention of these things are found in the corresponding medical file
Category E	Cause of death given heart failure. In the medical file heart failure is mentioned but it is also mentioned that the person died of renal failure. This changes the ICD-10 cause of death category
Category F	No (major) discrepancies

For both the formal and material requirements, a comparison was made between the medical specialists and the residents.

### Statistics

The independent samples t-test and Pearson's chi-squared test in IBM SPSS version 26 were used for comparing group means and proportions, respectively. A two-sided *P*-value < 0.05 was considered to indicate statistical significance.

## RESULTS

### General

**Figure 1.**
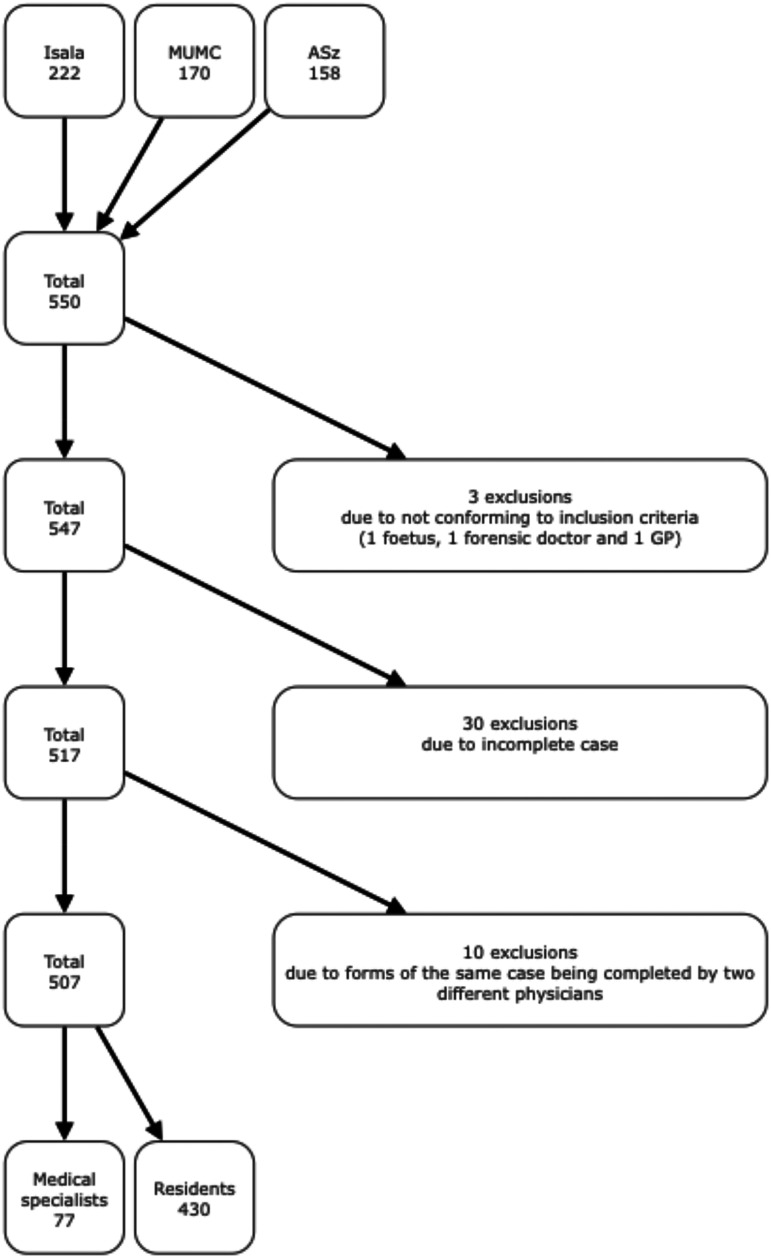
Inclusion of cases (Isala: Isala Hospital in Zwolle ; MUMC: Maastricht University Medical Centre+ in Maastricht; ASz: Albert Schweitzer Hospital in Dordrecht).

Overall, 550 cases were included. After the exclusion of 33 cases, 10 of which were excluded because the set of forms of the same case were unlawfully filled out by two different physicians, 507 cases were available for analysis (**Figure 1**).

### Formal Requirements

Most cases (83%) were filled out by residents (**Table 3**).

**Table 3.** CD, MCCD, and Envelopes Filled Out According to the Law.

	Medical specialists (n = 77)	Residents (n = 430)	Total (n = 507)	Difference between medical specialist and resident *(P-value)*
CD according to law	27 *(35%)*	168 *(39%)*	195 *(38.5%)*	*0*.*506*
MCCD according to law	35 *(45.5%)*	161 *(37.5%)*	196 *(38.7%)*	*0*.*506*
Envelope according to law	31 *(40%)*	155 *(36%)*	186 *(36.7%)*	*0*.*480*
CD, MCCD and envelope according to law	3 *(3.9%)*	31 *(7.2%)*	34 *(6.7%)*	*0*.*284*

Abbreviations: CD, certificate of death; MCCD, medical certificate of cause of death.

The CDs and MCCDs were not completed correctly or completely in 312 (61.5%) and 311 (61.4%) cases, respectively. Similarly, 321 of the MCCD envelopes (63.3%) were not filled out correctly or were not properly sealed. There were no statistically significant differences between medical specialists and residents on these points (**Table 3**).

In 89 cases (17.6%), both the CD and MCCD were correctly completed. Thirty-four cases (6.7%) met all the formal requirements including correctly filling out and sealing the MCCD envelope, and these proportions were not significantly different between medical specialists and residents (**Table 3**).

### Material Requirements

In 73% of the cases no major discrepancies between the cause of death on the MCCD and in the medical file were observed (Category F). The prevalence of the categories of mistakes in material requirements is presented in **Table 4**. There were no statistically significant differences between medical specialists and residents in the prevalence of material requirements mistakes (*P* *=* *0.063*, **Table 4**).

**Table 4.** Categories of Mistakes in the MCCD.

Categories	Medical specialists (n = 77)	Residents (n = 430)	Total (n = 507)
Category A (unnatural death)	1 *(1.3%)*	15 *(3.5%)*	16 *(3.1%)*
Category B (stated death not a cause of death)	0 *(0%)*	11 *(2.5%)*	11 *(2.2%)*
Category C (phenomenon)	9 *(11.7%)*	46 *(10.7%)*	55 *(10.8%)*
Category D (cause of death not found in file)	0 *(0%)*	12 *(2.8%)*	12 *(2.4%)*
Category E (different cause of death)	4 *(5.2%)*	38 *(8.8%)*	42 *(8.3%)*
Category F (no discrepancies)	63 *(81.8%)*	308 *(71.7%)*	371 *(73.2%)*

Abbreviation: MCCD, medical certificate of cause of death.

In only 23 cases (4.5%), all formal and material requirements for filling out the CD and MCCD and the envelope were met. This proportion was comparable between medical specialists (3 of 77, 3.9%) and residents (20 of 430, 4.6%) (*P* *=* *0.769)*.

## DISCUSSION

This study aimed to determine whether attending physicians in Dutch hospitals correctly and accurately filled out the CD and MCCD pertaining to deceased persons. Only 34 cases (6.7%) met all formal requirements, and in 136 cases (26.8%) major discrepancies between the stated cause of death on the MCCD and the medical file were found. Perhaps the most striking finding was that 16 cases (3.1%) documented as natural deaths were actually unnatural deaths. Altogether, only 4.5% of the cases (n = 23) fulfilled all formal and material requirements for correctly and accurately completed death certificates.

Errors in death certificates have been reported from countries across the world, including the United States (10,11), the Middle East (Saudi Arabia, Iran, Lebanon and Palestine) (4), Cyprus (12), East Asia (13–15), the UK (16,17), and Switzerland (18). Although most studies have focused on errors in the recorded cause of death, failure to meet formal requirements has also been mentioned. These included errors such as missing or incorrectly reported personal information of the deceased, absence of the time interval between onset of the cause of death and death or absence of the signature of the attending physicians. These errors were also observed in this study. The CD and the MCCD are official legal documents completed by the attending physician to certify a person's death and to testify to the cause of death. As mentioned previously when physicians sign the CD, they declare themselves to be convinced of a natural death. If the death is in fact of an unnatural cause, an incorrectly completed death certificate impedes any further postmortem investigations and may hamper criminal law and due process.

This study showed that 73% of the cases had no major discrepancies between the cause of death on the MCCD and in the medical file. Though this is better than the average of about 50% from other studies (4,11–19), it should be taken into account that this study merely looked at major discrepancies.

The accuracy of the MCCD is an indispensable source of mortality information. Consequently, the accuracy of the MCCD directly impacts the accuracy of the cause of death statistics in a country. This is relevant not only for the reliability of these statistics within and between countries, but also for reliable public health epidemiology. If causes of death are inaccurate, prevention may be focused on the wrong cause of death and therefore public funding might be misdirected.

Although our study does not provide any data on the reasons why death certificates were filled out incorrectly or inaccurately, we hypothesize that several factors may play a role. First, the registration burden for physicians has increased (19). Another factor could be the lack of a sense of urgency to fill out death certificates correctly and accurately. Most physicians tend to perceive their living patients as more important than their deceased patients and do not understand the importance of correct and accurate death certificates. The absence of the terms *postmortem examination*, *death*, and *death certificates* in the Dutch Medical Education Framework 2024 (20) demonstrates this. Filling out the appropriate form may be perceived as an added registration burden. The reason for the lack of knowledge of the importance of death certificates could be the lack of education regarding the subject. The previously mentioned international studies emphasize the role of education on the subject of death certificates (21). In Dutch medical curricula, both undergraduate and postgraduate, little if any education is provided on the subject of postmortem examination and the ensuing filling out of required forms. As this subject is not included in the Dutch Medical Training Framework (20), as mentioned before, it is not perceived as mandatory and as such as not important. This is reflected by the similar poor performance between medical specialists and residents in the present study. This finding is in agreement with earlier work showing that licenced medical specialists and general practitioners have similar knowledge and skills on postmortem examinations as residents (6,7). The alarming results of the present study call for more attention to the execution of external postmortem examinations and a careful and diligent completion of the accompanying forms for physicians in training. It could be advised that a dedicated and independent physician, for example a seconded forensic physician, checks the correctness and accuracy of the CDs and MCCDs completed in hospitals and who would report a case to the forensic physician on call if needed. In England a similar recommendation was made by the Home Office in the 2003 Home Office Fundamental Review of Death Certification and Investigation (22). The impact of a medical examiner was assessed in pilots across the UK (23). Starting in September 2024 all deaths in healthcare situations in England that are not investigated by a coroner will be reviewed by medical examiners (The Medical Examiners (England) Regulations 2024) (24). If such a system is implemented in the Netherlands with a role for the forensic physician this would have an impact on work of a forensic physician, who already faces substantial demands. Although there is currently a shortage of forensic physicians, the role and tasks of medical examiners as described in the English system, fit the activities and fall logically into the area of expertise, of a forensic physician in the Dutch system. The current dynamics of the forensic medical field in the Netherlands, with its orientation into task differentiation, could open up the possibility of adding the role of medical examiner, as described in the English system, to the tasks of the Dutch forensic physician.

As mentioned earlier, other studies on the subject of external postmortem examination also revealed potential differences between junior and more senior physicians and showed no significant difference between the two groups in terms of their consistency in acts and thoughts regarding external postmortem examination or in their legal knowledge on the subject (6,7). One of the obvious solutions to create awareness among undergraduate medical students regarding the importance of correctness and accuracy of completing death certificates, for example in combination with the legal aspects of death and dying. This should be followed by a more practical training during the postgraduate medical education where residents are trained, in theory and in practice, to fill out CDs and MCCDs. This could be done not only with a course on how to fill out the appropriate forms and how to use the International Classification of Diseases, tenth edition (ICD-10), for the cause of death but also with bedside teaching by knowledgeable medical specialists (21).

Given that more countries in and outside of Europe struggle with designing an effective system of postmortem investigation and filling out associated certificates and forms, it stands to reason to bundle efforts for a quality improvement and strive towards a single European standard (17).

## CONCLUSIONS

Hospital physicians seldom met the formal requirements for filling out the appropriate forms regarding a patient's death. In addition, major mistakes in the stated cause of death on the MCCD were found in one in four cases.

Correctness and accuracy of completing the death certificates are important not only from a legally administrative and criminal law point of view but also for public health epidemiology and related funding of prevention of the established causes of death. These results urgently call for more awareness, and careful and timely teaching regarding the importance and the skills of external postmortem examination and accurate completion of death certificates for medical students and residents. Another, but major, step that could be taken is to implement the use of medical examiners in the Netherlands, as is being done in England. It would stand to reason to add the role of medical examiner to the work of the Dutch forensic physician.
